# How Does Self-Concept Differentiation Work in Chinese Retirees: A Moderated Mediation Analysis

**DOI:** 10.3389/fpsyg.2021.555339

**Published:** 2021-02-18

**Authors:** Changzheng Zhu, Min Zhu, Xiangping Gao, Xiaoshi Liu

**Affiliations:** ^1^Department of Psychology, Shanghai Normal University, Shanghai, China; ^2^Department of Psychology, Henan University, Kaifeng, China; ^3^Department of Social Work and Management, Nanjing Tech University, Nanjing, China; ^4^Department of Psychology, Tsinghua University, Beijing, China

**Keywords:** self-concept differentiation, self-consistency and congruence, psychological adjustment, retirees, moderated mediation model

## Abstract

Self-concept differentiation (SCD) is a sign of fragmentation of the self rather than specialization of role identities for its robust relationship with psychological adjustment. However, little is known about the mechanisms underlying the relationship between SCD and psychological adjustment. The aim of this study was to examine the mediating role of self-consistency and congruence (SCC) in the association between SCD and psychological adjustment (psychological well-being, depression, and anxiety), and the moderating role of age in the relationship between SCD and SCC. This moderated mediation model was examined among 158 Chinese retirees (mean age = 71.12, *SD* = 9.13), who completed measurements regarding SCD, SCC, psychological well-being, anxiety, and depression. The results showed that SCC partially mediated the links between SCD and the indices of psychological adjustment. Furthermore, age moderated this mediation effect, which was found in mean and high-age participants, but not in low-age ones. Our findings indicate that, at different age stages, the internal mechanisms of SCD affecting psychological adaptation are not the same, and a low differentiated or highly integrated self can serve as an adaptive resource to maintain high subjective well-being of the elderly and protect them from anxiety and depression.

## Introduction

People have many social identities, and they can easily and smoothly switch among these identities. For example, Lisa is a strict teacher in a preschool but a tender wife and lenient mother at home. Those different identities would be internalized into self-concept (Francis and Adams, 2019). As a result, self-concept becomes more and more complicated and structured along with individuals’ socialization. Thus, self-concept is conceptualized as a multifaceted and dynamic cognitive structure (Donahue et al., 1993; Diehl et al., 2001), and the focus of studies has been moved from the content of self-concept to the self-structure in the past three decades. Up to now, there are several models of self-structure, such as self-complexity (Linville, 1985), self-compartmentalization (Showers, 1992), and self-concept differentiation (SCD; Donahue et al., 1993), that seek to explain how the components of the self-concept are organized and how structural-organizational features of self-concept are related to psychological adaptive function. The most widely studied among them is the model of SCD which refers to the degree to which an individual’s self is variable or consistent across personally important roles (Donahue et al., 1993; Diehl et al., 2001; Pilarska, 2017).

### Self-Concept Differentiation

The initial, also the biggest controversy over SCD, is whether the differentiation of the self is a sign of specialization or a fragmentation. Gergen (1971) argues that holding high levels of SCD means that individuals possess specialized identities that enable them to respond to different role requirements flexibly and adaptively. Contrary to Gergen, Donahue et al. (1993) interpret highly differentiated self-concept as a sign of fragmentation of the self and individuals with high levels of SCD could be seen as ones who possess a divided self and face many unresolved intrapsychic conflicts. This controversy intrigued researchers to examine the relationship between SCD and psychological adaptation. These researchers had found robust relationship patterns: SCD was negatively related to positive psychological adjustment, such as sense of well-being (Donahue et al., 1993; Diehl et al., 2001; Diehl and Hay, 2011), self-acceptance (Diehl et al., 2001), and self-esteem (Donahue et al., 1993; Bigler et al., 2001; Diehl et al., 2001; Lutz and Ross, 2003) and positively related to maladjustment, such as anxiety (Donahue et al., 1993; Bigler et al., 2001; Diehl et al., 2001; Peleg and Messerschmidt-Grandi, 2018), loneliness (Lutz and Ross, 2003), and depression (Donahue et al., 1993; Bigler et al., 2001; Diehl et al., 2001; Lutz and Ross, 2003; Diehl and Hay, 2011). Similar patterns of relationships between SCD and the measures of psychological adjustment also existed at different levels of personality, such as trait SCD and narrative SCD (Dunlop et al., 2013). These findings support that SCD is a sign of fragmentation of the self rather than specialization of role identities. Individuals who see themselves very differently across their roles tend to be more maladaptive than individuals who see themselves as similar across roles (Donahue et al., 1993; Pilarska, 2017). Low levels of SCD can be seen as resources to cope with pressure and challenges.

Though the relationship between SCD and measures of psychological adjustment has already been investigated, little research has been conducted on the mechanisms underlying this link. Dunlop et al. (2013) argued that future researchers on SCD should find the mediators in the relations between SCD and adjustment. Furthermore, they predicted that self-reported beliefs, such as clarity and consistency of self-concepts, may play the roles of mediators between SCD and adjustment. Pilarska (2016) has found that self-concept clarity mediates the relationship between SCD and sense of identity, i.e., SCD weakens sense of identity through reducing self-concept clarity. The finding suggested that holding diverse self-views would impede maintaining sense of identity by eliciting uncertainty and confusion about the self.

### Self-Consistency and Congruence

Rogers (1959) hypothesized that the self was a multifaceted structure which consisted of a number of self-aspects such as real self and ideal self. The notion of congruence vs. incongruence among the self-aspects was a central premise of Rogers’ theory. Individuals could be said to approach self-aspect congruence, or to be adjusted or fully functioning if their experience was closely aligned with their ideal self; however, individuals would be expected to experience maladjustment and psychological distress if they were to experience a substantial discrepancy among self-aspects, and they would be said to be incongruent (Rogers, 1959). Self-consistency and congruence (SCC) refers to an individual’s internal harmony and the coordination between self and experience, including self-evaluation ability, emotion, and self-congruity (Rogers, 1959). Therefore, SCC means the consistency of the inner self and the identification of oneself and experience (Gu et al., 2016). The researchers in China have found that SCC acts as a mediator of the relationship between personality dimensions and psychosomatic symptoms (Wang, 1994), and family atmosphere and loneliness (Yang et al., 2013). These findings suggested that the impact of intrapersonal or environmental variables on mental health and psychological adjustment may be due to their effects on SCC, which in turn affects psychological adjustment. Sheldon et al. (2012) examined the relationship among SCD, SCC, and subjective well-being and found that SCC was associated with SCD, and the effects of SCD on subjective well-being could be explained by SCC. Summarily, SCC, as perception and experience of the discrepancy among self-aspects, is an important mediator underlying the relationship between self-concept structure and psychological adjustment.

### Moderating Role of Age

Researchers also focus on how SCD changes with age. Developmental psychologists regard self as a cognitive and social structure, which differentiates and integrates constantly with the development of cognitive abilities (Labouvie-Vief et al., 1995; Harter, 2006, 2016; Esnaola et al., 2018). Diehl et al. (2001) described the age trajectory of SCD across the adult life span. They found that the relationship between age and SCD followed a curvilinear, U-shaped pattern. Specifically, from early adulthood to middle adulthood age, SCD tended to decrease, reaching its lowest level in late middle age. Conversely, from late adulthood to old age, SCD tended to increase. Hay and Diehl (2010) found that age difference in reactivity to daily stressors was moderated by SCD whereby the elderly with low levels of SCD were particularly resilient to home stressors. Furthermore, they assumed that similar levels of SCD are not necessarily evidences of equivalent mechanisms at different age.

According to Erikson’s psychosocial theory, the final stage of development is integrity vs. despair. Integrity is the major focus during the individual’s post-retirement, typically after age 65 (Hearn et al., 2012). Life review is a way to achieve self-integrity (Westerhof and Bohlmeijer, 2014) and is characterized by the progressive return to consciousness of past experiences and the resurgence of unresolved conflicts. Also, these revived experiences and conflicts could be surveyed and reintegrated (Butler, 1963; Wong and Watt, 1991). Life review also means a strong focus on self-experience and the differences in the self-structure which would induce self-disharmony (Westerhof et al., 2010). Taken together, we assumed that SCD could significantly predict SCC, but such predictive effects only happened in the older individuals who probably spent more time on life review.

### Purpose and Conceptual Models

Although there is a set of established associations between SCD and psychological adjustment, the underlying mechanisms of this relationship are unclear. A large body of previous studies has suggested that SCC, as perception and experience of the discrepancy among self-aspects, might be an important mediator underlying the effects of SCD on psychological adjustment (Sheldon et al., 2012). Thus, in the present study we aimed at examining the mediating effect of SCC in the association between SCD and psychological adjustment. Otherwise, we would like to check the moderation role of age on the mediating effect. To examine the moderated mediation model, in the current study we focused on Chinese retirees aged from 55 to 92.

**Figure 1:**
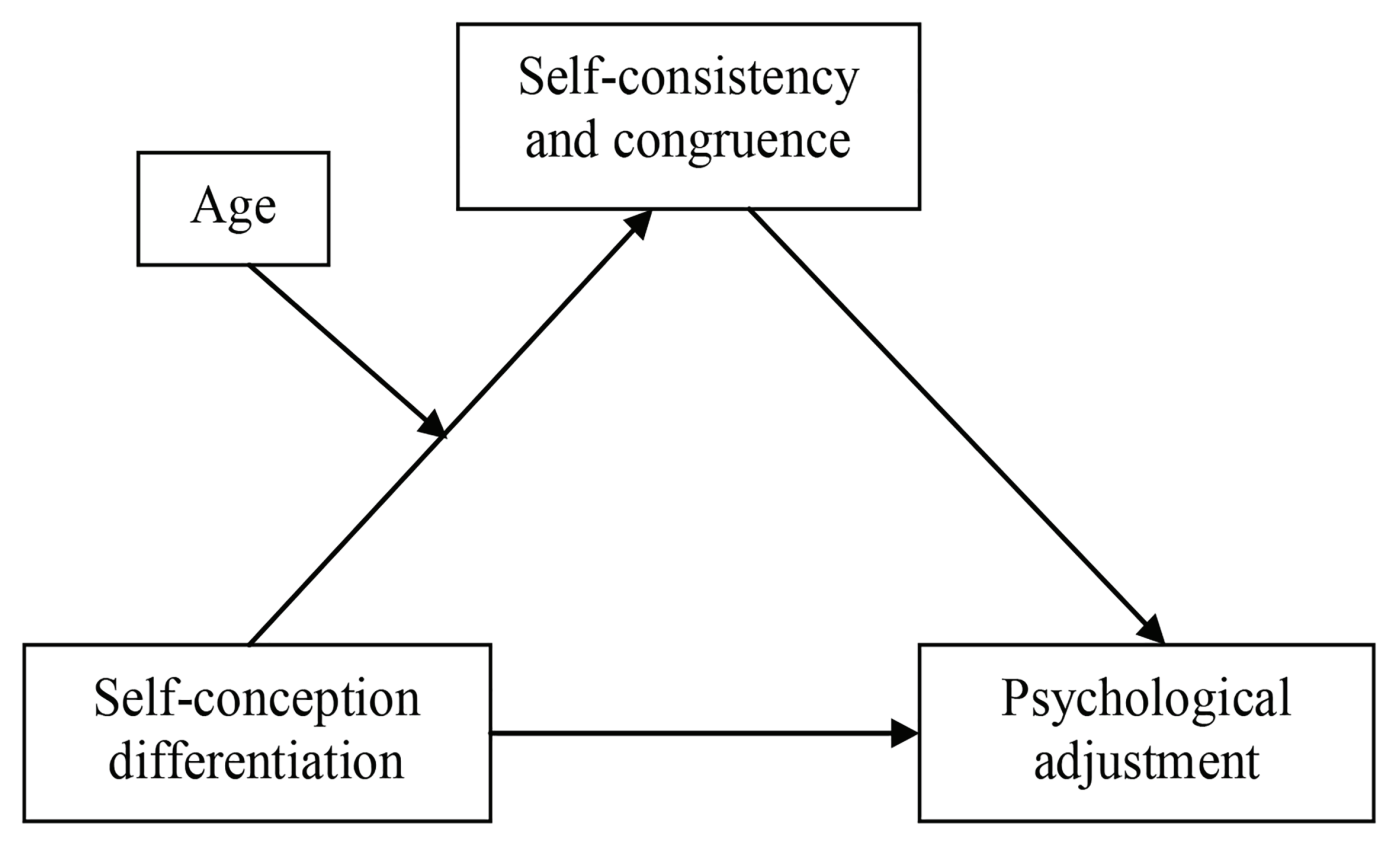
Theoretical models of moderated mediation effect linking self-concept differentiation and psychological adjustment.

As a summary, we proposed a moderated mediation model, in which SCC would mediate the relationship between SCD and psychological adjustment, and age would moderate the mediating effect of SCC in the first stage (Figure 1).

## Materials and Methods

### Participants

All of the participants were retirees from Henan University. One-hundred-eighty-seven questionnaires were administered; 177 of them were recollected. Exclusion of participants with incomplete data resulted in a final sample of 158 participants (81 men and 77 women).^1^ The valid returned rate was 84%. The age of the participants ranged from 55 to 92 years (*M* = 71.12 years, *SD* = 9.13 years). Thereinto, 51 of the participants (32%) were between the ages of 55 and 64 (*M* = 61.25, *SD* = 2.36), 47 of the participants (30%) were between the ages of 65 and 74 (*M* = 68.94, *SD* = 2.80), and the remaining 60 participants (38%) were over 75 (*M* = 81.40, *SD* = 3.91). The mean schooling years was 13.33 (*SD* = 3.46). Before retirement, 28 participants were professors or the equivalent; 55 participants were associate professors or the equivalent; 34 participants were lecturers or the equivalent; and the remaining 41 participants were others (teaching assistant or support staff). Thus, participants were divided into two groups: high social position (including professor or the equivalent and associate professor of the equivalent, total 83 participants) and low social position (including lecturer or the equivalent and others, total 75 participants). The study was approved by the Henan University Ethics Review Board.

### Procedure

Questionnaires were encapsulated in a portfolio and then taken home by participants or delivered to participants by liaisons. Liaisons are members of retirement administration of Henan University and responsible for contacting with retirees from this university. Participants were instructed to complete the questionnaires at their own pace at home (within 3 days at most) and sent back to researchers either by themselves or by the liaison. Each participant who completed the questionnaires was offered a cleaning supplies gift pack (facial tissue and laundry detergent) as a reward.

### Measures

#### Self-Conception Differentiation

Level of SCD was measured with the procedure originally developed by Block (1961), which was recently used by researchers such as Diehl and his coworkers (Diehl et al., 2001; Diehl and Hay, 2011). Participants were asked to rate their five different roles on 40 personality-descriptive attributes ranging from 1 (extremely uncharacteristic) to 8 (extremely characteristic). These roles, including parents, son or daughter, worker, friend, and partner, were familiar for participants and often were experienced in daily life. Participants who did not currently have one role were instructed to think of what they were like in those relationships in the past. The 40 personality-descriptive attributes (20 positives and 20 negatives) are broad personal characteristics which have been shown to be applicable to most of the social roles (Donahue et al., 1993), such as easy-going, talkative, cautious, and enthusiastic. The words used in our study were chosen from the Chinese adjective list, which is applicable to describe Chinese personality characteristics (Cui and Wang, 2003). Before participants rated every role, they were asked to recall and experience the situation when they played that role. Each role was presented on a separate page, and there were two orders in which the roles were presented to participants, and the measures in both orders have no significant difference, *t* (156) = 0.42, *p* = 0.67.

An index of SCD was derived from the subject’s ratings in the following procedure. For each participant, the ratings for the five roles were intercorrelated and the resulting 5 × 5 correlation matrix was factor analyzed. The first principle component, representing the variance shared across five roles identities, was extracted, and the remaining variance (100% minus the percentage of variance accounted for by the first principle component) was the index of SCD. Higher values indicate more unshared variance by the roles and therefore reflect higher levels of SCD. Such measure has been used in a number of studies, and its reliability and criterion validity has been established (Donahue et al., 1993; Bigler et al., 2001; Diehl et al., 2001; Lutz and Ross, 2003; Diehl and Hay, 2011).

#### Self-Consistency and Congruence Scale (Chinese Version)

Based on Rogers’ concept of SCC and his scale for measuring consistency between self and experience, Wang (1994) developed the SCCS, with a total of 35 items rated on a 5-point Likert-type scale ranging from 1 (strongly disagree) to 5 (strongly agree). The scale encompasses 3 subscales. Subscale-1 included 16 questions on self and experience disharmony (e.g., I feel that my current situation is far from what I expected); Subscale-2 had 12 questions on self-flexibility (e.g., I can change my mind if I am convinced by evidence); and Subscale-3 included 7 questions on self-stereotyping (e.g., I will not change idea once I have my own opinions). Total scores were computed such that higher scores represented more harmonious self. The measure has been used and proved reliable and valid (Sang et al., 2007; Yang et al., 2013; Gu et al., 2016; She et al., 2016). In this study, Cronbach’s *α* is 0.78.

#### Psychological Well-Being Scale

Psychological well-being scale (PWBS) is an 84-item self-rating inventory that assesses the six areas of psychological well-being (PWB): autonomy, environmental mastery, personal growth, positive relations with others, purpose in life, and self-acceptance (Ryff, 1989). Subjects were requested to rate their agreement with each item using a 6-point Likert-type scale ranging from 1 (strongly disagree) to 6 (strongly agree). A sample item from the PWBS is “For me, life has been a continuous process of learning, changing and growth.” Summative scores were computed such that higher scores represented higher PWB. The PWBS is suitable for different countries and has been shown to be reliable and valid (Ryff, 1989; Ruini et al., 2003). In this study, Cronbach’s *α* is 0.93.

#### Geriatric Depression Scale

Participants’ depressive symptoms were measured by administering a 30-item form of *The Geriatric Depression Scale* (GDS; Brink et al., 1982). Participants used “yes” or “no” to judge agreement with such items as “Do you feel pretty worthless the way you are now?” The highest possible score is 30, which indicates a very high depressive state. Developed to exclude the effects of nonspecific somatic symptoms such as anorexia and insomnia, GDS is a widely used screening tool for depression in older adults (Andrews et al., 2017) and has been proved reliable and valid (Brink et al., 1982; Andrews et al., 2017; Tarugu et al., 2019). In this study, Cronbach’s *α* is 0.75 State-Trait Anxiety Inventory, Form Y (STAI-Y). STAI-Y, developed by Spielberger (1985), consists of 40 items to be rated on a 4-point scale ranging from 1 (not at all true) to 4 (always true). A sample item is “My nerves are tense.” The measure assesses separate dimensions of “state” anxiety (items l–20) as well as “trait” anxiety (items 21–40). A total score was gained with higher scores indicative of greater anxiety. The STAI-Y has been found to be reliable and valid (Bergua et al., 2012). In this study, Cronbach’s *α* is 0.91.

## Results

### Common Method Bias

We employed Harman’s single-factor test to estimate the common method bias. By taking variables (SCC, PWB, anxiety, and depression) into an exploratory factor analysis and examining the unrotated factor solution, we found that there were 44 factors with eigenvalues larger than 1 and the first factor could account for 23.34% covariance among the measures, less than the critical value 40%, which showed that there were no common method biases in the study.

### Descriptive Statistics and Descriptive Analyses

The descriptive statistics and the bivariate correlations of the study variables are shown in Table 1.

**Table 1:** Means, SD, and correlations among variables.

	1	2	3	4	5	6	7	8
1. Age								
2. Gender	−0.24^**^							
3. Social position	−0.36^***^	0.34^***^						
4. SCD	0.01	0.00	0.16^*^					
5. SCC	−0.01	0.06	−0.090	−0.34^***^				
6. PWB	0.15	0.03	−0.26^**^	−0.38^***^	0.47^***^			
7. Anxiety	−0.01	0.00	0.15	0.38^***^	−0.46^***^	−0.57^***^		
8. Depression	0.12	0.08	0.07	0.30^***^	−0.38^***^	−0.32^***^	0.40^***^	
*M*	71.19	0.50	0.47	0.16	2.54	4.37	1.67	5.25
*SD*	9.13	0.50	0.50	0.13	0.31	0.38	0.31	3.54

Gender: 0, male, 1, female; social position: 0, high, 1, low; SCD, self-concept differentiation; SCC, self-consistency and congruence; and PWB, psychological well-being.

* *p* < 0.05;

** *p* < 0.01;

*** *p* < 0.001.

As shown in Table 1, the study variables were all significantly related to each other. SCD was negatively related to SCC, *r* = −0.34, *p* < 0.001. SCD was negatively related to positive psychological adjustment, such as PWB (*r* = −0.38, *p* < 0.001), and positively related to negative psychological adjustment, such as anxiety (*r* = 0.38, *p* < 0.001) and depression (*r* = 0.29, *p* < 0.001). SCC was positively related to positive psychological adjustment, such as PWB (*r* = 0.47, *p* < 0.001), and negatively related to negative psychological adjustment, such as anxiety (*r* = −0.48, *p* < 0.001) and depression (*r* = −0.38, *p* < 0.001). A demographic variable should be considered as a confounding variable and be included in the regression models as a covariate if it was significantly related to predictor as well as either to the mediator and/or outcome (MacKinnon et al., 2000). In this study, only social position met the criteria for inclusion in the regression models. The highest variance inflation factor value was 2.72, suggesting that there is no multicollinearity among predictor variables.

### Relationship Between SCD and Psychological Adjustment: A Moderated Mediation Model

The data were analyzed using Model 7 in the PROCESS macro for SPSS, Release 3.2 (Hayes, 2018). The predictor (SCD), mediator (SCC), moderator (age), and the outcome (PWB, anxiety, and depression) were all standardized using a z-transformation. Social position was entered into the model as a covariate. The moderated mediated model equations are:

$$SCC=a_{0}+a_{1}SCD+a_{2}Age+a_{3}SCD\times Age+e_{2}$$ (1)

$$Y=c_{0}^{\prime }+c_{1}^{\prime }SCD+b_{1}SCC+e_{2}$$ (2)

**Figure 2:**
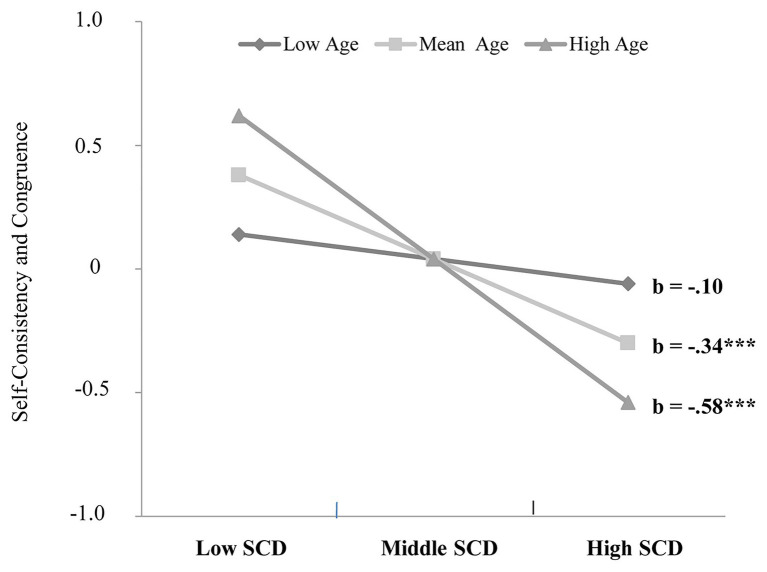
The moderated effect of age on the relationship between self-concept differentiation and self-consistency and congruence.

Equation (1) was tested, and the results showed that SCD significantly and negatively predicted SCC, *β* = −0.34 (0.07), *t* = −4.62, *p* < 0.001, 95% CI = [−0.491, −0.197], age did not significantly predict SCC, *β* = −0.02 (0.08), *t* = −0.25, *p* = 0.80, 95% CI = [−0.176, 0.136], and SCD × age of moderation effect was significant, *β* = −0.24 (0.07), *t* = −3.43, *p* < 0.001, 95% CI = [−0.380, −0.102]. Simple-slope analysis indicated that mean and high-age retirees showed a significant negative relation between SCD and SCC (*β* = −0.34, *t* = −4.62, *p* < 0.001; *β* = −0.58, *t* = −5.60, *p* < 0.001), but low-age retirees showed no relation between these two constructs (*β* = −0.10, *t* = −1.03, *p* = 0.304). The results support our hypothesis that the relationship between SCD and SCC is moderated by age and that SCD has no effects on SCC for low-age participants, but for mean and high-age participants, SCD is significantly and negatively associated with SCC. There is a significant trend in the relationship between SCD and SCC: along with the increasing of the age, the impact of SCD on SCC is enhanced gradually (Figure 2).

PWB, anxiety, and depression as the response variables entered Equation (2), respectively. SCD significantly predicted all of the indices of psychological adjustment, PWB, *β* = −0.22 (0.07), *t* = −3.05, *p* < 0.01, 95% CI = [−0.361, −0.077], anxiety, *β* = 0.24 (0.07), *t* = 3.28, *p* < 0.01, 95% CI = [0.096, 0.388], and depression, *β* = 0.19 (0.08), *t* = 2.39, *p* < 0.05, 95% CI = [0.033, 0.344]. SCC significantly predicted all of indices of psychological adjustment, PWB, *β* = 0.38 (0.07), *t* = 5.33, *p* < 0.001, 95% CI = [0.238, 0.519], anxiety, *β* = −0.37 (0.07), *t* = −5.03, *p* < 0.001, 95% CI = [−0.513, −0.224], and depression, *β* = −0.31.24 (0.08), *t* = −3.98, *p* < 0.001, 95% CI = [−0.464, −0.156].

The conditional indirect effect of SCD on the indices of psychological adjustment was calculated by the formula: $\;M=\left(a_{1}+a_{3}Age\right)\;\;\times \;\;b_{1}$. The results are presented in Table 2. For all of three indices of psychological adjustment, the indirect effect of SCD in the condition of low age was not significant. The indirect effect of SCD in the condition of mean and high age was all significant.

**Table 2:** Results for conditional indirect effect of self-concept differentiation on psychological adjustment through self-consistency and congruence.

Depending variable	Direct effect	Variable	Age	Indirect effect (SE)	95% CI
Psychological well-being	−0.22	Low age (−1 SD)	62.06	−0.04 (0.06)	[−0.183, 0.045]
		Average age	71.19	−0.13 (0.05)	[−0.250, −0.054]
		High age (+1 SD)	80.32	−0.22 (0.07)	[−0.360, −0.100]
Anxiety	0.24	Low age (−1 SD)	62.06	0.04 (0.05)	[−0.048, 0.168]
		Average age	71.19	0.13 (0.04)	[0.052, 0.229]
		High age (+1 SD)	80.32	0.22 (0.06)	[0.090, 0.339]
Depression	0.19	Low age (−1 SD)	62.06	0.03 (0.04)	[−0.044, 0.126]
		Average age	71.19	0.11 (0.04)	[0.038, 0.184]
		High age (+1 SD)	80.32	0.18 (0.06)	[0.060, 0.301]

Pairwise contrasts between conditional indirect effects (Effect1 minus Effect2) indicated that the effects at high age are greater than the same effects at mean age, PWB, contrast = −0.09, *E* = 0.04, 95% CI = [−0.158, −0.013], anxiety, contrast = 0.09, *E* = 0.04, 95% CI = [0.010, 0.162], and depression, contrast = 0.07, *E* = 0.04, 95% CI = [0.009, 0.152], and greater than the same effects at low age, PWB, contrast = −0.18, *E* = 0.07, 95% CI = [−0.317, −0.026], anxiety, contrast = 0.18, *E* = 0.08, 95% CI = [0.019, 0.3242], and depression, contrast = 0.15, *E* = 0.07, 95% CI = [0.018, 0.305]; the effects at mean age are also greater than the same effects at low age, PWB, contrast = −0.09, *E* = 0.04, 95% CI = [−0.158, −0.01], anxiety, contrast = 0.09, *E* = 0.04, 95% CI = [0.010, 0.162], and depression, contrast = 0.07, *E* = 0.04, 95% CI = [0.009, 0.152]. The results showed that the conditional indirect effects of SCD on psychological adjustment through SCC at various levels of age are significantly different from each other. The bootstrapped 95% CI did not include 0 for the pairwise contrasts between conditional indirect effect, which further confirmed that the mediation effect is moderated by age.

**Figure 3:**
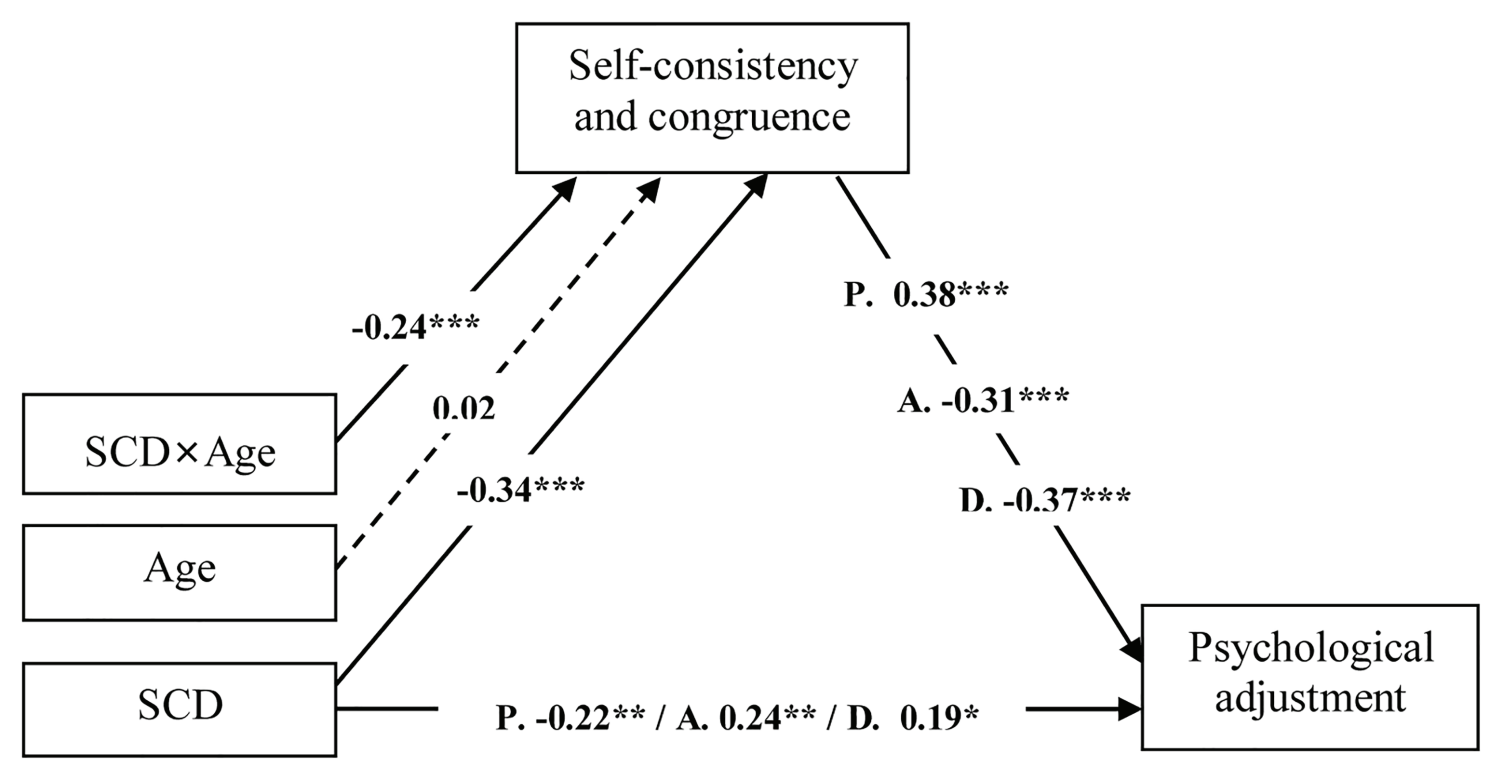
Moderated mediation effect linking SCD and psychological adjustment. Path coefficients “P” were for analysis using psychological well-being as a response variable. Path coefficients “A” were for analysis using anxiety as a response variable. Path coefficients “D” were for analysis using depression as response variable. Solid lines indicate pathways with significant parameter estimates. Dashed line indicates nonsignificant pathway. SCD, self-concept differentiation; ^*^p < 0.05, ^**^p < 0.01, ^***^p < 0.001.

Moderated mediation analysis has established that SCC mediates the relationship between SCD and psychological adjustment, and age moderates the mediating effect of SCC in the first stage (Figure 3).

## Discussion

The present study tested a theoretical model exploring both mediating (i.e., SCC) and moderating (i.e., age) factors in the links between SCD and psychological adjustment in a sample of Chinese retirees. Results indicated that (a) SCD was associated with psychological adjustment (e.g., high SCD, high depression and anxiety, and low PWB); (b) SCD was associated with SCC (e.g., high SCD and low SCC); (c) SCD was also indirectly linked with psychological adjustment *via* its negative association with SCC; and (d) these indirect paths were, in turn, moderated by age, such that the negative association between SCD and SCC was reinforced with aging. Taken together, the moderating mediation model suggested that the effects of SCD on psychological adjustment had different mechanisms at different ages. In the late middle age, there was no significant mediation effect and SCD was directly associated with psychological adjustment. The mechanisms of the relationship between SCD and psychological adjustment changed significantly in the old age, as the relationship between SCD and psychological adjustment was partially mediated by SCC.

### Implications of SCD for Retirees in China

The results of this study showed that SCD is negatively related to positive psychological adjustment (i.e., PWB) and positively related to negative psychological adjustment (i.e., anxiety and depression). Consistent with SCD’s purported role of risk factor (Donahue et al., 1993; Dunlop et al., 2013), we have found that for Chinese retirees higher differentiation of self-concept is associated with poor psychological adjustment, which indicates that high level of SCD is a sign of fragmentation rather than specialization of role identities. The impact of SCD is universal either in Eastern or Western cultures.

Given the developmental changes in the old age, such as the declining of mental and physical functioning, role losses, and chronic health problems, which are experienced as aversive and more or less inevitable, it is not surprising that researchers have made pessimistic assumptions about older people’s lives (Seligman, 1975; Hamm et al., 2020). However, growing evidences have indicated that this portrayal of old age is biased. The aging self, as an adaptive resource, can effectively help the elderly cope with various developmental changes and maintain a sense of control and a positive view of self and personal development (Brandtstädter and Greve, 1994). Lang (2017) suggested that the stability and continuity of self-representations may reflect a powerful psychological and cognitive adaptation of the human mind, which functionally operates even in the face of dramatic loss and health decline until very late in life. The relationship between SCD and psychological adjustment found in the current study implies that a highly integrated or low-differentiated self may serve as an adaptive resource to maintain a high subjective well-being and protect Chinese retirees from anxiety and depression. Aging self, or rather, the integrated self, is an adaptive resource for the elderly.

### SCC Mediated the Relationship Between SCD and Psychological Adjustment

SCD is a structural aspect of the self and can be defined as the extent to which a person’s self-concept varies across social roles or situations (Donahue et al., 1993; Diehl et al., 2001). As an index of the differentiation of self-structure, SCD mirrors the extent of differentiation or integration of self-concept and is independent of the specific content of self-concept (personal attributes) and self-aspects (Donahue et al., 1993). In contrast, SCC, as a type of psychological experience or feeling of the self, may be defined as the extent to which a person perceives congruence or incongruence among self-aspects (Rogers, 1959), which derives from the differences in the structure of self-concept, but is not itself a structural feature of the self-concept. Consistent with Sheldon et al. (2012), the current study found that SCD had a moderate negative association with SCC, that is, the more differences exist among components of self-structure, the less congruence an individual perceives among them. Holding diverse self-views (high levels of SCD) may elicit the sense of incongruence and inconsistency. These findings indicate that SCD and SCC are two distinct but closely related variables. SCD, as the underlying structure of self, influences psychological experience of the self.

Previous studies showed that SCC mediated the relationship between intrapersonal or environmental variables and psychological adjustment (Wang, 1994; Yang et al., 2013). Dunlop et al. (2013) predicted that consistency of self-concepts may mediate the association between SCD and adjustment. Consistent with Sheldon et al. (2012), the present study found that SCD was indirectly linked with psychological adjustment *via* its negative association with SCC. The mediation model inferred that the impact of the organization of self-aspects on psychological adjustment may be due to its effects on SCC, which in turn affects psychological adjustment. The low differentiation in the self-structure makes individuals feel that they are consistent and congruent, which is beneficial to the improvement of positive psychological adaptation (i.e., PWB) and the reduction of negative psychological adaptation (i.e., anxiety and depression). On the contrary, high differentiation in self-concept makes individuals feel contradictions and conflicts, which breeds negative psychological adaptation and reduces positive psychological adaptation.

### Age Moderated the Mediation Effect of SCC

The current study examined the moderated mediation model: SCC partially mediated relations between SCD and indices of psychological adjustment, and age moderated this mediation effect, such that the negative association between SCD and SCC was reinforced with aging. The mediation effect was found in mean and high age retirees, but not in low-age ones. The moderated mediation model suggested that the effects of SCD on psychological adjustment had different mechanisms at different age stages. There was no significant mediation effect in the late middle age, and SCD affected psychological adjustment directly. However, SCC partially mediated the relationship between SCD and psychological adjustment, and the mediation effect increased significantly with aging.

The different mechanisms could be interpreted by the disengagement theory (Tornstam, 1989; Hamm et al., 2020) and the development view of late life of Erikson (1982). Disengagement theory is an influential theory of old age, in which successful aging is defined as a mutual withdrawal of the aging individual and the social system. As “Growing Old,” the ability of the elderly inevitably declines and they generally have a decreasing trend of being involved in the social world. Meanwhile, Erikson proposes that there is an increasing trend of introversion in old ages. In this study, participants were chosen from Henan University retirees, ranging in age from 55 to 92 years. Late-middle-aged participants have just left their jobs and were actually in a “working state.” Some were rehired by Henan University to work as teaching supervisors; some were actively involved in learning activities organized by the university for retirees, such as calligraphy, art, music, and photography, to make up their previously neglected interests; and at the same time almost all of them threw themselves into the work of looking after their grandchildren. This busy and colorful retirement stopped them from reviewing and integrating their past experiences, and they could not be fully aware of the differences in self-structure, which resulted in the insignificant mediating effect of SCC in the low-age group. With the increase of age and the decline of physical function, older individuals gradually withdrew from various social activities, and they began to enter the real retirement life. Thus, the senior would have more time with themselves to look into their inner world and look back to their past experiences. Life review would enable the senior to recall different aspects of the self at different ages simultaneously. In the process of life review, the inconsistencies and even the conflicts underlying the structure of the self would probably be more and more frequently perceived by the senior. As a result, the extent of differentiation or integration of self-aspects is more likely to affect their sense of self-harmony and further affect their psychological adaptation.

### Limitation and Future Inquiries

The present study provided some first evidence of the underlying mechanisms that may help to interpret the relationship between SCD and psychological adjustment in Chinese adults (ranging in age from 55 to 90). Limitations should be noted with the current study, with an eye toward future directions. First, participants in the current study were asked to complete a series of questionnaires, in which the measurement of SCD required participants to have strong reading comprehension. Considering the difficulty with the questionnaire and the convenience of sampling, the sample was drawn from Henan University retirees with higher level of education. The specificity of the sample limited the size of the sample and reduced the external validity of the results (Finkenzeller et al., 2019). Future studies should expand the breadth and size of samples to improve the external validity of the results. Second, the current study found that the meditation effect of SCC was moderated by age. However, age is a special independent variable which cannot be manipulated. Also, from Jung’s and Erikson’s theoretical perspective on the development of late life, it is not age itself but changes with age (e.g., the decreasing trend of being involved in the social world and the increasing trend of introversion and life review) that act as moderators. Future studies should consider new moderating variables, such as the duration and frequency of involvement in the social world and introversion.

## Data Availability Statement

The raw data supporting the conclusions of this article will be made available by the authors, without undue reservation.

## Ethics Statement

The studies involving human participants were reviewed and approved by The ethics committee of Henan University. The patients/participants provided their written informed consent to participate in this study.

## Author Contributions

CZ, MZ, XG, and XL designed the experiments. CZ and MZ recruited the participants, collected the data, and prepared the draft. CZ performed the data analysis. XG and XL reviewed it critically and gave important intellectual content. All authors contributed to the article and approved the submitted version.

### Conflict of Interest

The authors declare that the research was conducted in the absence of any commercial or financial relationships that could be construed as a potential conflict of interest.

## Footnotes

^1^We calculated the post power (1-*β*) according to linear multiple regression: fixed model, *R*^2^ increase by the *post hoc* power analysis in the G-power (Faul et al., 2007). Effect size *f*^2^ = 0.22 was calculated by moderation effect *R*^2^ = 0.18 (from the moderation effect of age between SCD and SCCS). By using an alpha of 0.05, total sample size of 158, and 1 tested predictor, the final power (1-*β*) was calculated as 0.99.

## References

[ref1] Andrews J. A. Astell A. J. Brown L. J. E. Harrison R. F. Hawley M. S. (2017). Technology for early detection of depression and anxiety in older people. Stud. Health Technol. Inform. 242, 374–380. 10.3233/978-1-61499-798-6-374, PMID: 28873826

[ref2] Bergua V. Meillon C. Potvin O. Bouisson J. Amieva H. (2012). The stai-y trait scale: psychometric properties and normative data from a large population-based study of elderly people. Int. Psychogeriatr. 24, 1163–1171. 10.1017/S1041610212000300, PMID: 22436140

[ref3] Bigler M. Neimeyer G. J. Brown E. (2001). The divided self revisited: effects of self-concept clarity and self-concept differentiation on psychological adjustment. J. Soc. Clin. Psychol. 20, 396–415. 10.1521/jscp.20.3.396.22302

[ref4] Block J. (1961). Ego identity, role variability, and adjustment. J. Consult. Psychol. 25, 392–397. 10.1037/h0042979, PMID: 13870048

[ref5] Brandtstädter J. Greve W. (1994). The aging self: stabilizing and protective processes. Dev. Rev. 14, 52–80. 10.1006/drev.1994.1003

[ref6] Brink T. L. Yesavage J. A. Lum O. Heersema P. H. Adey M. Rose T. L. (1982). Screening tests for geriatric depression. Clin. Gerontol. 1, 37–43. 10.1300/J018v01n01_06 7183759

[ref7] Butler R. N. (1963). The life review: an interpretation of reminiscence in the aged. Psychiatry 26, 65–76. 10.1080/00332747.1963.11023339, PMID: 14017386

[ref8] Cui H. Wang D. F. (2003). The confirmation of Chinese personality structure and the result of adjective ratings. Stud. Psychol. Behav. 1, 89–95.

[ref9] Diehl M. Hastings C. T. Stanton J. M. (2001). Self-concept differentiation across the adult life span. Psychol. Aging 16, 643–654. 10.1037/0882-7974.16.4.643, PMID: 11766918

[ref10] Diehl M. Hay E. L. (2011). Self-concept differentiation and self-concept clarity across adulthood: associations with age and psychological well-being. Int. J. Aging Hum. Dev. 73, 125–152. 10.2190/AG.73.2.b, PMID: 22010361 PMC3198817

[ref11] Donahue E. M. Robins R. W. Roberts B. W. John O. P. (1993). The divided self: concurrent and longitudinal effects of psychological adjustment and social roles on self-concept differentiation. J. Pers. Soc. Psychol. 64, 834–846. 10.1037/0022-3514.64.5.834, PMID: 8505712

[ref12] Dunlop W. L. Walker L. J. Wiens T. K. (2013). What do we know when we know a person across contexts? Examining self-concept differentiation at the three levels of personality. J. Pers. 81, 376–389. 10.1111/jopy.12018, PMID: 23126485

[ref13] Erikson E. (1982). The life cycle completed: A review. New York: Norton.

[ref14] Esnaola I. Sesé A. Antonio-Agirre I. Azpiazu L. (2018). The development of multiple self-concept dimensions during adolescence. J. Res. Adolesc. 30, 100–114. 10.1111/jora.12451, PMID: 30156745

[ref15] Faul F. Erdfelder E. Lang A. G. Buchner A. (2007). G*power 3: a flexible statistical power analysis program for the social, behavior, and biomedical sciences. Behav. Res. Methods, 39, 175–191. dio 10.3758/BF03193146, PMID: 17695343

[ref16] Finkenzeller T. Pötzelsberger B. Kösters A. Würth S. Amesberger G. Dela F.. (2019). Ageing in high functioning elderly persons: study design and analyses of behavioral and psychological factors. Scand. J. Med. Sci. Sports 29, 7–16. 10.1111/sms.13368, PMID: 30570174 PMC6850373

[ref17] Francis L. E. Adams R. E. (2019). Two faces of self and emotion in symbolic interactionism: from process to structure and culture-and back. Symb. Interact. 42, 250–277. 10.1002/symb.383

[ref18] Gergen K. J. (1971). The concept of self. New York: Holt, Rinehart and Winston, Inc.

[ref19] Gu Y. M. Hu J. Hu Y. P. Wang J. R. (2016). Social supports and mental health: a cross-sectional study on the correlation of self-consistency and congruence in China. BMC Health Serv. Res. 16:207. 10.1186/s12913-016-1463-x, PMID: 27353410 PMC4924263

[ref20] Hamm J. M. Heckhausen J. Shane J. Lachman M. E. (2020). Risk of cognitive declines with retirement: who declines and why? Psychol. Aging 35, 1–16. 10.1037/pag0000453, PMID: 32175753 PMC7165065

[ref21] Harter S. (2006). “The self” in Handbook of child psychology: Social, emotional, and personality development. Vol. 3. eds. Damon W. Lerner R. M. (New York: Wiley), 505–570.

[ref22] Harter S. (2016). “I-self and me-self processes affecting developmental psychopathology and mental health” in Developmental psychopathology, theory and method. 3rd Edn. ed. Cicchetti D. (Hoboken, NJ: John Wiley & Sons).

[ref23] Hay E. L. Diehl M. (2010). Reactivity to daily stressors in adulthood: the importance of stressor type in characterizing risk factors. Psychol. Aging 25, 118–131. 10.1037/a0018747, PMID: 20230133 PMC2841317

[ref24] Hayes A. F. (2018). Introduction to mediation, moderation, and conditional process analysis second edition: A regression-based approach. New York, NY: Guilford Press.

[ref25] Hearn S. Saulnier G. Strayer J. Glenham M. Koopman R. Marcia J. E. (2012). Between integrity and despair: toward construct validation of Erikson’s eighth stage. J. Adult Dev. 19, 1–20. 10.1007/s10804-011-9126-y

[ref26] Labouvie-Vief G. Chiodo L. M. Goguen L. A. Diehl M. (1995). Representations of self across the life span. Psychol. Aging 10, 404–415. 10.1037/0882-7974.10.3.404, PMID: 8527061

[ref27] Lang F. R. (2017). “Age, self, and identity: structure, stability, and adaptive function” in Encyclopedia of geropsychology. ed. Pachana N. A. (Singapore: Springer), 122–131.

[ref28] Linville P. W. (1985). Self-complexity and affective extremity: don't put all of your eggs in one cognitive basket. Soc. Cogn. 3, 94–120. 10.1521/soco.1985.3.1.94

[ref29] Lutz C. J. Ross S. R. (2003). Elaboration versus fragmentation: distinguishing between self-complexity and self-concept differentiation. J. Soc. Clin. Psychol. 22, 537–559. 10.1521/jscp.22.5.537.22927

[ref30] MacKinnon D. P. Krull J. L. Lockwood C. M. (2000). Equivalence of the mediation, confounding and suppression effect. Prev. Sci. 1, 173–181. 10.1023/A:1026595011371, PMID: 11523746 PMC2819361

[ref31] Peleg O. Messerschmidt-Grandi C. (2018). Differentiation of self and trait anxiety: a cross-cultural perspective. Int. J. Psychol. 54, 816–827. 10.1002/ijop.12535, PMID: 30289168

[ref32] Pilarska A. (2016). How do self-concept differentiation and self-concept clarity interrelate in predicting sense of personal identity? Personal. Individ. Differ. 102, 85–89. 10.1016/j.paid.2016.06.064

[ref33] Pilarska A. (2017). Effects of self-concept differentiation on sense of identity: the divided self revisited again. Pol. Psychol. Bull. 48, 255–263. 10.1515/ppb-2017-0029

[ref34] Rogers C. R. (1959). “A theory of therapy, personality, and interpersonal relationship as developed in the client-centered framework” in Psychology: A study of science. ed. Koch S. (New York: McGraw Hill).

[ref35] Ruini C. Ottolini F. Rafanelli C. Tossani E. Ryff C. D. Fava G. A. (2003). The relationship of psychological well-being to distress and personality. Psychother. Psychosom. 72, 268–275. 10.1159/000071898, PMID: 12920331

[ref36] Ryff C. D. (1989). Happiness is everything, or is it? Explorations on the meaning of psychological well-being. J. Pers. Soc. Psychol. 57, 1069–1081. 10.1037/0022-3514.57.6.1069

[ref37] Sang Q. S. Ge M. G. Yao Q. (2007). The relationship of college students' self-consistency and congruence with life stress and life satisfaction. Psychol. Sci. 30, 552–554. 10.1016/S1874-8651(08)60032-0

[ref38] Seligman M. E. P. (1975). Helplessness: on depression, development, and death. San Francisco: WH Freeman & Co.

[ref39] She P. Zeng H. Yang B. (2016). Effect of self-consistency group intervention for adolescents with schizophrenia: an inpatient randomized controlled trial. J. Psychiatr. Res. 73, 63–70. 10.1016/j.jpsychires.2015.11.006, PMID: 26688437

[ref40] Sheldon K. M. Gunz A. Schachtman T. R. (2012). What does it mean to be in touch with oneself? Testing a social character model of self-congruence. Self Identity 11, 51–70. 10.1080/15298868.2010.503130

[ref41] Showers C. (1992). Compartmentalization of positive and negative self-knowledge: keeping bad apples out of the bunch. J. Pers. Soc. Psychol. 62, 1036–1049. 10.1037/0022-3514.62.6.1036, PMID: 1619548

[ref42] Spielberger C. D. (1985). Assessment of state and trait anxiety: conceptual and methodological issues. Southern Psychologist 2, 6–16.

[ref43] Tarugu J. Pavithra R. Vinothchandar S. Basu A. Chaudhuri S. John K. R. (2019). Effectiveness of structured group reminiscence therapy in decreasing the feelings of loneliness, depressive symptoms and anxiety among inmates of a residential home for the elderly in Chittoor district. Int. J. Community Med. Public Health 6, 847–854. 10.18203/2394-6040.ijcmph20190218

[ref44] Tornstam L. (1989). Gero-transcendence: a reformulation of the disengagement theory. Aging Clin. Exp. Res. 1, 55–63. 10.1007/BF03323876 2488301

[ref45] Wang D. F. (1994). Compilation of self-consistency and congruency scale (SCC) (in Chinese). J. Clin. Psychol. 2, 19–22.

[ref46] Westerhof G. J. Bohlmeijer E. T. (2014). Celebrating fifty years of research and applications in reminiscence and life review: state of the art and new directions. J. Aging Stud. 29, 107–114. 10.1016/j.jaging.2014.02.003, PMID: 24655678

[ref47] Westerhof G. J. Bohlmeijer E. Webster J. D. (2010). Reminiscence and mental health: a review of recent progress in theory, research and interventions. Ageing Soc. 30, 697–721. 10.1017/S0144686X09990328

[ref48] Wong P. T. Watt L. M. (1991). What types of reminiscence are associated with successful aging? Psychol. Aging 6, 272–279. 10.1037/0882-7974.6.2.272, PMID: 1863396

[ref49] Yang L. Tong J. Miao S. Wuhan Psychological Hospital (2013). Influences of family atmosphere on loneliness: the mediation of self-consistency. J. Health Psychol. 21, 1725–1729. 10.13342/j.cnki.cjhp.2013.11.015

